# IMPOWER: a national patient-generated registry for intestinal malrotation exploring diagnosis, treatment, and surgical outcomes

**DOI:** 10.1186/s13023-023-02722-5

**Published:** 2023-05-11

**Authors:** Sydney A. Martinez, Scott C. Fligor, Savas Tsikis, Meagan Short, Katie E. Corcoran, Amy Rogers, Kathleen M. Gura, Mark Puder

**Affiliations:** 1 University of Oklahoma Health Sciences Center, 801 NE 13th St., Oklahoma City, OK 73104 USA; 2 Vascular Biology Program and the Department of Surgery, Boston Children’s Hospital, Boston, MA 02115 USA; 3 Harvard Medical School, Boston, MA 02115 USA; 4 Intestinal Malrotation Foundation, Arrington, TN 37014 USA; 5 West Virginia University, 29 Beechurst Ave, Morgantown, WV 26505 USA; 6 Department of Pharmacy and Division of Gastroenterology, Hepatology, and Nutrition, Boston Children’s Hospital, Boston, MA 02115 USA

**Keywords:** Intestinal malrotation, Volvulus, Rare disease registry program, Ladd procedure, Short bowel syndrome

## Abstract

**Background:**

Intestinal malrotation is a rare congenital condition with potentially devastating consequences due to potential volvulus and massive intestinal necrosis. Diagnosis is often delayed and long-term symptoms following surgical correction are poorly characterized. We developed the Intestinal Malrotation Patient Outcomes and WEllness Registry (IMPOWER), a national patient-generated registry (PGR), to capture data related to presenting symptoms, testing, diagnosis, treatment, and follow-up of individuals diagnosed with malrotation. IMPOWER captures patient-reported information from adult patients and parents/caregivers of children diagnosed with malrotation at the time of enrollment and at ongoing 6-month intervals. We present baseline characteristics of patients enrolled during the first two months of the registry.

**Results:**

Within the first two months, 354 patients with malrotation enrolled in IMPOWER, and 191 (53.9%) completed all baseline assessments. Nearly 90% of the 119 pediatric participants and 37.7% of the 72 adult participants experienced symptoms prior to diagnosis. Vomiting was the predominant symptom for pediatric participants compared to abdominal pain in adults. Yellow bilious emesis was more commonly reported than green, and volvulus at diagnosis occurred in 70% of pediatric and 27% of adult participants. One-third of pediatric participants had a bowel resection as part of their initial surgical procedure, resulting in 23.4% with diagnosed short bowel syndrome. More than 60% of pediatric and 80% of adult registrants reported gastrointestinal symptoms that persisted throughout the first year following their initial operation. Approximately 25% of registrants reported visiting four or more gastroenterologists for management of ongoing symptoms.

**Conclusions:**

Fewer than half of pediatric patients presented with the “classic” presentation of green bilious colored emesis. Yellow bilious emesis was more commonly reported, and chronic gastrointestinal symptoms (i.e., abdominal pain, reflux, constipation, diarrhea) and feeding intolerance were common following surgical procedures for malrotation. This novel PGR highlights the need for a multicenter prospective registry to characterize the natural history and develop consistent standards of care related to the diagnosis, treatment, and long-term care for patients with malrotation.

**Supplementary Information:**

The online version contains supplementary material available at 10.1186/s13023-023-02722-5.

## Background

Intestinal malrotation encompasses a broad spectrum of rare congenital anomalies of intestinal rotation and fixation estimated at one in 500 births, based on a case series of 2000 patients with gastrointestinal symptoms undergoing radiographic examination of the colon [1]. The incidence of malrotation, however, is likely underestimated as many patients remain asymptomatic. Symptomatic malrotation is thought to occur in one out of every 6000 live births [2], although population-based studies have found rates of 2.86 per 10,000 live births and fetal deaths [3] and rates of 0.28–0.35 per 1000 live births [4]. The majority of cases of symptomatic malrotation are diagnosed in neonates or infants, although individuals can be diagnosed with malrotation at any age [5–7]. Due to the rarity of malrotation, there is often a delay in identifying and recognizing symptoms, diagnosis, and surgical treatment. This delay is particularly devastating in cases of intestinal volvulus, given the possibility for extensive bowel necrosis which can result in short bowel syndrome or death [6, 8–11].

The Ladd procedure is considered definitive surgical management for intestinal malrotation. Persistent gastrointestinal symptoms among patients with malrotation following this procedure are thought to be uncommon [6]. However, social media support groups describe a constellation of persistent postoperative gastrointestinal symptoms including feeding intolerance, constipation, abdominal pain, and subsequent surgical procedures, which may impact quality of life. These postoperative symptoms have not been studied systematically.

Since intestinal malrotation is a rare disease, hospital-based studies are generally limited by small sample sizes and may be underpowered to describe outcomes and natural history adequately. A potential solution to this problem is a Patient-Generated Registry (PGR), which is a disease registry created, maintained, and populated by patients themselves to generate hypotheses and inform the development of future research studies [12]. PGRs are supported by the National Institutes of Health, with resources in their Rare Disease Registry Program [13], and the Agency for Healthcare Research and Quality. [14] Patient-reported outcomes are emerging as an important tool for health outcomes research.

We developed the Intestinal Malrotation Patient Outcomes and WEllness Registry (IMPOWER), a national online PGR that captures data on symptoms, testing, diagnosis, treatment, and follow-up of individuals diagnosed with malrotation. This paper describes the development of this registry and presents baseline characteristics of patients enrolled in the first two months of recruitment. With the IMPOWER PGR, we aim to inform the development of a multi-institutional prospective clinical registry to improve patients’ diagnosis, treatment, and outcomes of malrotation.

## Methods

### Study design

IMPOWER is an online PGR that captures patient-reported information from adult patients and parents/caregivers of children diagnosed with malrotation. The registry includes a comprehensive baseline assessment at enrollment and ongoing six-month follow-up surveys. IMPOWER was designed at the University of Oklahoma Health Sciences Center in collaboration with the Intestinal Malrotation Foundation (IMF) and the Department of Surgery at Boston Children's Hospital. An IMPOWER Advisory Board was established, consisting of researchers, medical professionals, and IMF patient representatives to advise the development and maintenance of the registry, dissemination of research findings, and future decisions related to data use. The IMPOWER Advisory Board developed assessments and pilot tested the registry with patients before launch. The University of Oklahoma Health Sciences Center Institutional Review Board approved the protocol and procedures. (IRB#12681).

### Study population and recruitment

IMF first announced the development of IMPOWER to various malrotation online patient communities in December 2020 and enrollment opened on January 1^st^, 2021. Participants were recruited using online social media platforms and websites targeted at disease-specific patient communities. These included patient Facebook groups (Awareness for Malrotation, Children with Malrotation and Volvulus, IMF), the IMF Instagram page, and the IMF website [15–17]. The registry launched prior to the Intestinal Malrotation and Volvulus Awareness Day on January 15th. It was promoted extensively in January and February, leading up to Rare Disease Day on February 28th. Participants who enrolled from January 1st to March 5th 2021, were included in this analysis.

Participants were eligible to enroll if they were 18 years or older, resided in the USA, read English, and had a valid email address. Participants were eligible if they self-reported they were either (1) the parent/guardian of a child currently under the age of 18 previously diagnosed by a clinician with intestinal malrotation or (2) previously diagnosed with intestinal malrotation themselves.

## Data collection

### Screening/Informed consent/enrollment

Study data were collected and managed using REDCap electronic data capture tools hosted at the University of Oklahoma Health Sciences Center [18, 19]. REDCap (Research Electronic Data Capture) is a secure, web-based application designed to support data capture for research studies, providing: (1) an intuitive interface for validated data entry; (2) audit trails for tracking data manipulation and export procedures; (3) automated export procedures for seamless data downloads to common statistical packages, and (4) procedures for importing data from external sources.

Online recruitment information included the link to a self-administered screening form. Eligible participants were prompted to provide a current email address and received an email invitation and unique registration link. Participants were then directed to additional information and consent forms. Adult participants and parents/guardians of pediatric participants were required to provide electronically signed informed consent. Parents enrolling their child aged 7 to 17 were required to report they informed their child about the registry and obtain the child’s verbal assent. Child assent was not required for children currently under the age of seven. Once informed consent was completed, participants entered their baseline health information using a series of REDCap surveys (See Additional file 1, which presents the surveys).

### Measures

Patient demographics included age, gender, sex, state of residence, race, and ethnicity. For symptoms and diagnosis, participants reported their location of residence, age of diagnosis, and symptoms at diagnosis. Participants reported length of time from symptoms to diagnosis, occurrence of urgent care visits, required emergency care, or hospitalizations for gastrointestinal symptoms before diagnosis, test procedures leading up to diagnosis, and the test procedure and clinician type that confirmed the diagnosis.

Participants were asked if they had received a surgical procedure for malrotation and/or volvulus and were asked to provide details related to the timing and type of surgical intervention, length of hospital stay, presence of bowel resection, and types of medical management present at discharge. Participants also completed a postsurgical outcomes survey to report any surgical complications experienced immediately postoperatively and details related to any ongoing gastrointestinal symptoms within the first year following the surgical procedure. Regardless of surgical status, all participants were asked about current and past medical management of gastrointestinal symptoms. Participants were then offered the opportunity to agree to the 6-month follow-up survey and remain on a contact list for future research opportunities.

A one-time bereavement survey was also included for parents to register a deceased child. Parents reported symptoms before diagnosis, whether their child had a surgical procedure for malrotation and/or volvulus, surgery timing, bowel resection occurrence and length of intestinal loss, date of death, and whether death was related to malrotation.

### Statistical analysis

Descriptive statistical analyses were performed to summarize demographics and clinical data. Proportions were calculated for categorical measures. Means and standard deviations, as well as medians and ranges, were calculated for age. Statistical analyses were conducted using the SAS Version 9.4 software (SAS Institute Inc, Cary, NC, USA).

## Results

Between January 1 through March 5, 2021, a total of 354 participants began enrollment, and 191 (53.9%) completed all IMPOWER baseline assessment forms. This included 119 (62.3%) pediatric and 72 (37.7%) adult participants. Due to differences in reporting and history of disease between adults who reported a childhood diagnosis and adults who reported their diagnosis in adulthood, we excluded 13 adult participants who registered and self-reported a childhood diagnosis and treatment from main analyses (Data not shown, see Additional file 2, which provides a comparison of adults by age at diagnosis). Table 1 displays patient demographics by type of registrant, comparing pediatric registrants compared to adult registrants diagnosed at 20 years of age or older. Ten pediatric registrants were deceased at the time of registration. At the time of enrollment, the median age was 4.4 years (range 0–17.5) for pediatric registrants and 38.3 years (range 22.6–66.7) for adult registrants.

**Table 1:** Participant demographics and clinical characteristics in IMPOWER^a^

Characteristic	Pediatric registrants^b^ (n = 119)	Adult registrants diagnosed after 20 years of age (n = 55)
*Age at enrollment, years*		
Mean (SD)	5.7 (4.4)	40.2 (10.6)
Median (range)	4.4 (0–17.5)	38.3 (22.6–66.7)
*Age category at enrollment, n(%)*		
< 1	12 (10.3)	–
1 to < 9	82 (70.1)	–
9 to < 18	23 (19.7)	–
18 to < 44	-	38 (69.1)
44 +	-	17 (30.9)
Female, n(%)	52 (43.7)	51 (92.7)
White Race, n(%)	110 (94.0)	55 (100.0)
*Region*		
Northeast	18 (15.1)	14 (25.4)
Midwest	28 (23.5)	17 (30.9)
South	46 (38.7)	15 (27.3)
West	15 (12.6)	9 (16.4)
*Age at diagnosis, n(%)*		
< 1 month	68 (57.1)	–
1 month to < 1 year	22 (18.4)	–
1 year to < 5 years	15 (12.6)	–
5 years to < 10 years	9 (7.6)	–
10 years to < 20 years	4 (3.4)	–
20 years or more	–	55 (100.0)
Symptoms present prior to diagnosis, n(%)	106 (89.1)	54 (98.2)
*Time from symptoms to diagnosis, n(%)*		
Under 1 month	65 (54.6)	6 (10.9)
1 month or more to less than 6 months	22 (18.5)	6 (10.9)
6 months or more to less than 1 year	2 (1.7)	3 (5.5)
1 year to less than 5 years	10 (8.4)	8 (14.5)
5 years or more	6 (5.0)	31 (56.4)
No symptoms or unknown	14 (11.7)	1 (1.8)
*All symptoms reported prior to diagnosis, n(%)*		
Vomiting	86 (72.3)	28 (50.9)
Excessing spit up as a baby	64 (53.8)	7 (12.7)
Lethargy/weakness	47 (39.5)	28 (50.9)
Other	44 (37.0)	22 (40.0)
Abdominal pain	43 (36.1)	51 (92.7)
Constipation	38 (31.9)	37 (67.3)
Swollen abdomen	34 (28.6)	31 (56.4)
Pallor/pale in color	32 (26.9)	12 (21.8)
Failure to thrive	26 (21.8)	7 (12.7)
Diarrhea	13 (10.9)	27 (49.1)
*Primary symptom at diagnosis, n(%)*		
Vomiting	46 (38.7)	5 (9.1)
Excessing spit up as a baby	18 (12.6)	0 (0.0)
Lethargy/weakness	3 (2.5)	1 (1.8)
Other	18 (15.1)	6 (10.9)
Abdominal pain	10 (8.4)	36 (65.5)
Constipation	1 (0.8)	1 (1.8)
Swollen abdomen	8 (6.7)	3 (5.5)
Pallor/pale in color	1 (0.8)	0 (0.0)
Failure to thrive	3 (2.5)	0 (0.0)
Diarrhea	1 (0.8)	2 (3.6)
*Volvulus at diagnosis, n(%)*		
Yes	83 (69.7)	15 (27.3)
No	28 (23.5)	34 (61.8)
*Green or yellow colored emesis, n(%)*		
Vomit/Spit Up Ever Green	55 (46.2)	12 (21.8)
Vomit/Spit Up Ever Yellow	70 (58.8)	24 (43.6)
Vomit/Spit Up Ever Red/Brown	12 (10.1)	4 (7.3)
Yellow spit up shortly after birth	52 (43.7)	–
*Pain constant or intermittent, n(%)*		
Constant	7 (5.9)	9 (16.4)
Intermittent	29 (24.4)	42 (76.4)
No pain or unknown	83 (69.8)	4 (7.3)

^a^Intestinal Malrotation Patient Outcomes and WEllness Registry

^b^Pediatric registrants under 18 at time of enrollment were registered by parent or caregiver

### Pediatric registrants

Among pediatric registrants, 57.1% were diagnosed with malrotation at less than one month of age (Table 1). Additionally, 18.4% were diagnosed after one month of age but less than one year. Nearly 90% had symptoms before diagnosis, with vomiting or excessive spit-up, lethargy/weakness, and abdominal pain most commonly reported. Only 46.2% reported green bilious emesis before diagnosis, while 58.8% reported yellow bilious emesis before diagnosis, and 43.7% reported yellow-tinted spit up as a newborn while in the hospital shortly after birth. Around 70% reported volvulus at diagnosis.

Pediatric registrants reported frequent healthcare visits before diagnosis, which included frequent pediatrician visits (44.5%) and emergency department visits (35.3%) (Table 2). When examining test procedures leading up to diagnosis, 73.9% of pediatric registrants received an upper gastrointestinal series (UGI) with or without a small bowel follow-through, while only half reported that the UGI confirmed the diagnosis. Close to 20% were confirmed at the time of surgery or autopsy. The confirmatory test was most often made by an emergency department clinician (27.7%) followed by a surgeon (18.5%). When assessing the treatment of intestinal malrotation, 89.9% of pediatric registrants reported undergoing a surgical procedure for malrotation and/or volvulus.

**Table 2:** Health care visits and testing until diagnosis among participants in IMPOWER^a^

Characteristic	Pediatric registrants^b^ (n = 119)	Adult registrants diagnosed after 20 years of age (n = 55)
*Healthcare visits prior to diagnosis*		
Frequent primary care/pediatrician visits	53 (44.5)	29 (52.7)
Specialty care visits	23 (19.3)	28 (50.9)
Urgent care visits	18 (15.1)	15 (27.3)
Emergency department visits	42 (35.3)	34 (61.8)
Hospitalizations	27 (22.7)	10 (18.2)
None of the above or unknown	40 (33.6)	7 (12.7)
*All tests leading up to diagnosis*		
Upper GI with small bowel follow through	63 (52.9)	24 (43.6)
X-ray	79 (66.4)	28 (50.9)
Ultrasound	48 (40.3)	29 (52.7)
CT	32 (26.9)	49 (89.1)
Upper GI	25 (21.0)	12 (21.8)
Lower GI/barium enema	16 (13.4)	11 (20.0)
MRI	15 (12.6)	17 (30.9)
Swallow study	14 (11.8)	6 (10.9)
Endoscopy	8 (6.7)	27 (49.1)
Gastric emptying study	7 (5.9)	12 (21.8)
Other test	14 (11.7)	13 (23.6)
No test, through surgery or autopsy	14 (11.8)	2 (3.6)
*Test that confirmed diagnosis*		
Upper GI with small bowel follow through	49 (41.2)	16 (29.1)
X-ray	11 (9.2)	2 (3.6)
Ultrasound	6 (5.0)	0 (0.0)
CT	13 (10.9)	38 (69.1)
Upper GI	11 (9.2)	0 (0.0)
Lower GI/barium enema	6 (5.0)	0 (0.0)
MRI	3 (2.5)	3 (5.5)
Swallow study	4 (3.4)	1 (1.8)
Endoscopy	1 (0.8)	1 (1.8)
Gastric emptying study	0 (0.0)	1 (1.8)
Other test	8 (6.7)	0 (0.0)
No test, through surgery or autopsy	23 (19.3)	5 (9.1)
*Clinician that ordered confirmation test*		
Emergency Department clinician	33 (27.7)	17 (30.9)
Surgeon	22 (18.5)	7 (12.7)
Intensive care unit (ICU) clinician	16 (13.4)	0 (0.0)
Primary care/pediatrician	15 (12.6)	10 (18.2)
Gastroenterologist	15 (12.6)	19 (34.5)
Other health care provider	10 (8.4)	0 (0.0)
Unknown	5 (6.9)	2 (3.6)
Surgical procedure for malrotation and/or volvulus	107 (89.9)	45 (81.8)

^a^Intestinal Malrotation Patient Outcomes and WEllness Registry

^b^Pediatric registrants under 18 at time of enrollment were registered by parent or caregiver

The majority of initial surgical procedures were reported as emergent to preserve life (Table 3). Approximately one-third underwent a bowel resection as part of their initial surgical procedure for malrotation, and 23.4% were diagnosed with short bowel syndrome following the initial surgical procedure. Surgical complications were reported by 15.9% of pediatric participants immediately following their initial surgical procedure. Within the first year of the initial surgical procedure, almost half of pediatric registrants had subsequent emergency department visits, and a third had additional hospitalizations. Approximately a quarter had additional surgical procedures within the first year, with 8.4% reporting three or more additional surgical procedures.

**Table 3:** Post-Surgical Outcomes among Surgical Participants in IMPOWER^a^

Characteristic, n(%)	Pediatric registrants^bc^ (n = 107)	Adult registrants diagnosed after 20 years of age (n = 45)
*Type of surgical procedure*		
Surgical intervention for volvulus	12 (11.2)	2 (4.4)
Surgical intervention for volvulus and Ladd’s	53 (49.5)	8 (17.8)
Ladd’s surgical procedure only	33 (30.8)	26 (57.8)
Alternative intestinal surgical procedure	4 (3.7)	9 (20.0)
*Urgency of surgical procedure*		
Emergency to preserve life	73 (68.2)	13 (28.9)
Urgent within 48 h	18 (16.8)	5 (11.1)
Recommended within next month	11 (10.3)	18 (40.0)
Elective or presented as a choice	2 (1.9)	9 (20.0)
Bowel resection during initial surgical procedure	34 (31.8)	10 (22.2)
Short bowel diagnosis after surgical procedure	25 (23.4)	3 (6.7)
*Length of hospital stay at initial surgical procedure*		
Under 1 day / outpatient	0 (0.0)	5 (11.1)
1 day or more but less than 3 days	7 (6.2)	10 (22.2)
3 days or more but less than 5 days	11 (9.7)	5 (11.1)
5 days or more but less than 10 days	32 (29.9)	13 (28.9)
10 days or more but less than 20 days	23 (21.5)	10 (22.2)
20 days or more	33 (30.8)	1 (2.2)
Postsurgical complications	17 (15.9)	13 (28.9)
*Outcomes after initial surgical procedure*		
Emergency department visits	52 (48.6)	27 (60.0)
Additional hospitalizations	35 (32.7)	15 (33.3)
Additional abdominal surgical procedure	27 (25.2)	14 (31.1)
Bowel obstruction	17 (15.9)	12 (26.7)
Volvulus or recurrent volvulus	3 (2.8)	3 (6.67)
*Number of additional surgical procedures*		
1 additional procedure	11 (10.3)	5 (11.1)
2 additional procedures	7 (6.5)	5 (11.1)
3 or more additional procedures	9 (8.4)	4 (8.9)

^a^Intestinal Malrotation Patient Outcomes and WEllness Registry

^b^Pediatric participants under 18 at time of enrollment were registered by parent or caregiver

^c^Excludes participants deceased at time of registration

When examining ongoing gastrointestinal symptoms following the initial surgical procedure, 62.6% of pediatric registrants reported gastrointestinal symptoms that persisted throughout the first year (Table 4). The most commonly reported symptoms were vomiting followed by difficulty tolerating foods, with each symptom reported in at least 20% of patients. Approximately 15% and 26% of pediatric registrants reported either the same or greater symptom severity or frequency after surgery, respectively. In the first year after the initial surgical procedure, 69.2% of pediatric patients visited at least one gastroenterologist, with 17% reporting visiting four or more gastroenterologists. Over 40% of patients visited a nutritionist/dietitian. At the time of registration, over a quarter of pediatric registrants were currently using medications (i.e., laxatives, antidiarrheal, motility, and pain medications), and a similar proportion utilized dietary changes to manage gastrointestinal symptoms.

**Table 4:** Ongoing gastrointestinal (GI) symptoms among surgical participants 1 year post operation in IMPOWER^a^

Characteristic, n(%)	Pediatric registrants^bc^ (n = 107)	Adult registrants diagnosed after 20 years of age (n = 45)
*GI symptoms reported 1 year following surgical procedure*		
Yes	67 (62.6)	37 (82.2)
No	38 (35.5)	7 (15.6)
*Type of GI symptoms 1 year following surgical procedure*		
Vomiting	39 (36.4)	11 (24.4)
Difficulty tolerating foods	36 (33.6)	19 (42.2)
Reflux	35 (32.7)	9 (20.0)
Abdominal pain	34 (31.8)	33 (73.3)
Constipation	30 (28.0)	22 (48.9)
Diarrhea	28 (26.2)	16 (35.6)
Excessive spit up (baby)	26 (24.3)	–
Swollen abdomen	26 (24.3)	13 (28.9)
Failure to thrive	21 (19.6)	5 (11.1)
Other	10 (9.3)	11 (24.4)
*Severity of symptoms 1 year following surgical procedure*		
More severe	7 (6.5)	10 (27.0)
About the same severity	9 (8.4)	9 (24.3)
Less severe	37 (34.6)	17 (46.0)
Generally no symptoms prior to surgical procedure	46 (43.0)	1 (2.7)
*Frequency of symptoms 1 year following surgical procedure*		
More frequent	10 (9.3)	13 (35.1)
About the same frequency	18 (16.8)	8 (21.6)
Less frequent	25 (23.4)	14 (37.8)
Generally no symptoms prior to surgical procedure	47 (43.9)	1 (2.7)
*Specialist visits following surgical procedure*		
Gastroenterologist	74 (69.2)	37 (82.2)
Nutritionist/Dietician	45 (42.1)	16 (35.6)
Physical therapist	29 (27.1)	5 (11.1)
Occupational therapist	27 (25.2)	–
Speech pathologist	25 (23.4)	–
Feeding therapist	22 (20.6)	–
Lactation specialist	17 (15.9)	–
Allergist	12 (11.2)	2 (4.4)
Other specialist	19 (17.8)	4 (8.9)
*Number of gastroenterologists seen following surgical procedure*		
0 gastroenterologists	33 (30.8)	8 (17.8)
1 gastroenterologist	20 (18.7)	10 (22.2)
2 gastroenterologists	27 (25.2)	8 (17.8)
3 gastroenterologists	9 (8.4)	3 (6.7)
4 or more gastroenterologists	18 (16.8)	16 (35.6)
*Nonsurgical methods currently used to manage symptoms*		
Medications	28 (26.2)	32 (71.1)
Dietary changes	27 (25.2)	31 (68.9)
Alternative medicine	6 (5.6)	10 (22.2)
Physical therapy	3 (2.8)	10 (22.2)
Other nonsurgical methods	12 (11.2)	3 (6.7)

^a^Intestinal Malrotation Patient Outcomes and WEllness Registry

^b^Pediatric participants under 18 at time of enrollment were registered by parent or caregiver

^c^Excludes participants deceased at time of registration

### Adult registrants

Among adult registrants aged 20 years or older at the time of reported diagnosis, 98.2% experienced symptoms before diagnosis (Table 1). Abdominal pain was overwhelmingly the most commonly reported symptom, along with constipation and swollen abdomen. Intermittent abdominal pain was the most prevalent (76.4%). Like pediatric registrants, adults were more likely to report yellow bilious emesis (43.6%) than green bilious emesis (21.8%). Adult registrants were likely to report frequent emergency department visits, specialty care visits, and primary care visits before diagnosis, and the confirmatory test for adults was most often a computed tomography (CT) (69.1%) ordered by a gastroenterologist or emergency department clinician (Table 2). Slightly lower than for pediatric patients, 81.8% of adult registrants reported undergoing a surgical procedure for malrotation.

Adult registrants most often reported the surgical procedure to be recommended within the next month (40.0%) or considered elective (20.0%) (Table 3). Surgical complications were reported by 28.9% of adult participants immediately following their initial surgical procedure. A third had additional surgical procedures within the first year, with 8.9% reporting three or more additional surgical procedures.

Over 80% of adults reported gastrointestinal symptoms that persisted throughout the first year following their initial surgical procedure (Table 4). The most commonly reported symptoms for adult participants were abdominal pain followed by constipation and difficulty tolerating foods. Over half of adults reported their symptoms to be more or just as severe or frequent as before surgery. In the first year after the initial surgical procedure, 82.2% of adult patients visited at least one gastroenterologist, and 35.6% reported visiting four or more gastroenterologists.

## Discussion

To our knowledge, this is the first PGR for intestinal malrotation and one of very few studies examining persistent gastrointestinal symptoms following diagnosis and surgery. Existing literature on malrotation often focuses on diagnosis in the acute setting (particularly of malrotation with volvulus), surgical treatment, and surgical outcomes–namely complications and the rate of recurrent volvulus. The prevalence of chronic symptoms in patients with malrotation and the resulting impact on quality of life are poorly understood. In this cohort, a significant proportion of patients suffered from chronic gastrointestinal symptoms before diagnosis of malrotation, and many patients continued to have persistent gastrointestinal symptoms despite ‘definitive’ surgical treatment for malrotation (i.e., the Ladd procedure).

This study highlights a critical need to further characterize the presenting symptoms of malrotation to reduce time to diagnosis, particularly in patients without classic symptoms (i.e., bilious emesis and acute abdomen in a child). One-quarter of the pediatric registrants and 65% of adult registrants in IMPOWER reported experiencing symptoms more than one year before diagnosis. Furthermore, nearly half of registrants reported frequent primary care visits related to gastrointestinal symptoms before diagnosis. Half of the pediatric registrants and 85% of adult registrants reported previous urgent care or emergency department visits for those symptoms. Previous studies of malrotation emphasize green bilious emesis as a classic and common symptom of intestinal malrotation with volvulus [9, 20, 21]. In contrast, we found the color of emesis/spit up was more variable and a yellow color was more common than green (58.8% vs. 46.2% in pediatric patients). Adult patients were also more likely to present with abdominal pain than emesis. Volvulus at diagnosis was more common in pediatric (69.7%) compared to adult registrants (27.3%). This suggests the need for further research to thoroughly document the variable clinical presentation of malrotation in the adult, pediatric, and infant patient populations.

The majority of previous studies examining Ladd procedure outcomes focus on recurrent volvulus and surgical outcomes, such as postoperative small bowel obstruction [22–25]. A contemporary multicenter retrospective cohort study of pediatric patients that underwent a Ladd procedure reported a 6% incidence of postoperative volvulus, 10% had a postoperative bowel obstruction, and 17% required an additional abdominal procedure [26]. Few studies investigate other postoperative complications or ongoing gastrointestinal symptoms [23, 25, 27, 28]. One retrospective chart review at a single institution examined resolution of symptoms and estimated that up to 89% of patients who underwent surgical intervention experienced symptom resolution [6]. However, the study methods, timeline of ascertaining outcome variables, and definition of resolution of symptoms through chart review were not well described.

In IMPOWER, we found that chronic gastrointestinal symptoms following a surgical procedure for malrotation were common. Approximately 63% of pediatric and 82% of adult registrants reported ongoing symptoms within the first year after their initial surgical procedure. Vomiting, feeding intolerance, reflux, and abdominal pain were each reported by over 30% of pediatric participants after their initial surgical procedure, whereas abdominal pain, constipation, and feeding intolerance were each reported by over 40% of adults. Our results also indicate that patients seek specialty care, including gastroenterology, nutrition, and feeding therapy at high rates following their initial surgical procedure. In particular, approximately 70% of pediatric and 82% of adult registrants reported seeking specialty care from gastroenterologists, with 17% of pediatric and 36% of adult registrants reporting seeing four or more gastroenterologists for ongoing symptoms. Our findings were similar to those of a recent 30-year study at two institutions which reported the presence of digestive symptoms including abdominal pain, reflux, nausea/vomiting, bloating, constipation, and diarrhea following the Ladd procedure as part of a newly defined clinicopathologic gut malrotation syndrome [29]. These recent findings suggest that patients often have ongoing gastrointestinal symptoms and feeding issues that are not relieved by surgical intervention and suggest that a referral to a gastroenterologist should be considered following the Ladd procedure in many patients.

The IMPOWER PGR has several limitations. First, the population recruited is likely not representative of all patients with intestinal malrotation. Recruitment occurred primarily from support groups that are likely comprised of participants with either chronic unaddressed needs or more complicated courses, which results in selection bias. Like other studies that recruited rare disease patients via social media, Caucasian participants and women appear to be overrepresented [30–32]. Facebook and Instagram users are disproportionately female [33], potentially accounting for the disparate recruitment. Second, PGRs are based on self-reported data. IMPOWER participants provided their own or their child’s medical information, and data were not validated through medical records. The registry questions were carefully designed and pre-tested with community members before launching the registry to partially overcome this limitation. This helped ensure that questions were clear and understandable to the malrotation community to reduce errors in responses. Despite these limitations, the data are valuable for identifying new hypotheses and establishing a cohort of potential study participants for future research on intestinal malrotation.

## Conclusions

Currently, intestinal malrotation is viewed as a congenital anomaly that presents acutely and undergoes definitive surgical intervention to minimize the risk of subsequent volvulus. The IMPOWER PGR demonstrates that diagnosis is often delayed, preceded by longstanding gastrointestinal symptoms. Following initial ‘definitive’ surgical treatment, many patients continue to have gastrointestinal symptoms, seek specialty care, and experience subsequent emergencies, hospitalizations, and surgical procedures. Thus, routine gastroenterology referral should be considered for all patients following surgery. As IMPOWER is a longitudinal registry, future studies will examine additional patients accumulated over time and long-term outcomes with serial follow-up surveys. This registry highlights the need to develop a multicenter prospective registry to further characterize the natural history of this rare disease. The data generated could be invaluable in establishing consistent standards of care related to diagnosis, treatment, and long-term care of patients with intestinal malrotation. It could also further establish the importance of a multidisciplinary team approach including surgery, gastroenterology, and nutrition to achieve optimal quality of life.

## Supplementary Information

**Additional file 1**. IMPOWER Questionnaires. This file presents the questionnaires used for the IMPOWER PGR

**Additional file 2**. **Tables S1–S4**. This file presents data from the adult registrants diagnosed before 20 years of age in comparison to the pediatric registrants and the adult registrants diagnosed after 20 years of age.

## Data Availability

The datasets used and/or analyzed during the current study are available from the corresponding author on reasonable request.

## Abbreviations

CT: Computed tomography
IMF: Intestinal Malrotation Foundation
IMPOWER: Intestinal Malrotation Patient Outcomes and WEllness Registry
PGR: Patient-Generated Registry
REDCap: Research Electronic Data Capture
UGI: Upper gastrointestinal series
