# Supplementary material for: IMPOWER: a national patient-generated registry for intestinal malrotation exploring diagnosis, treatment, and surgical outcomes
Source: Orphanet J Rare Dis. 2023 May 11;18:113. doi: 10.1186/s13023-023-02722-5 (PMC10176693; doi:10.1186/s13023-023-02722-5)
Supplement: Supplementary file 1 — Additional file 1. IMPOWER Questionnaires. This file presents the questionnaires used for the IMPOWER PGR [file 13023_2023_2722_MOESM1_ESM.pdf]

Additional File 2: IMPOWER Questionnaires

Patient Demographics

This section of the registry gathers information about the PATIENT or individual with malrotation, whether that is YOU or YOUR CHILD. Please provide the following information about the PATIENT with malrotation:

First Name:

Last Name:

Date of Birth:

Gender: 1=Female 2=Male 3=Prefer not to say

Sex assigned at birth: 1=Female 2=Male 3=Prefer not to say

State of residence:

Race (check all that apply):
1=White
2=Black or African American
3=American Indian or Alaska Native
4=Asian
5=Native Hawaiian or Other Pacific Islander

Ethnicity: 1=Hispanic or Latino 2=Not Hispanic

[If parent/guardian] Is your child currently living or deceased?
1=Living
2=Deceased

Symptoms and Diagnosis

This section of the survey asks about you or your child's symptoms and testing leading up to the malrotation diagnosis.

Were there any symptoms prior to diagnosis? 1=Yes 0=No

[If yes] What symptoms were present prior to diagnosis? (check all that apply)
1=Vomiting
2=Abdominal pain
3=Excessive spit up (as a baby)
4=Failure to thrive
5=Constipation
6=Diarrhea
7=Swollen abdomen
8=Pale in color
9=Lethargy or weakness
10=Other
11=I don't know

[If vomiting or spit up] Was the vomiting or spit up ever green? 1=Yes 2=No 3= I don't know

[If vomiting or spit up] Was the vomiting or spit up ever yellow? 1=Yes 2=No 3= I don't know

[If abdominal pain] Was the pain constant or intermittent (off and on)? 1=Constant 2=Intermittent (off and on) 3=I don't know
[If Other] Please specify "Other" symptoms:

What was the primary symptom (choose only one) leading to the test and/or diagnosis of malrotation?
1=Vomiting
2=Abdominal pain
3=Excessive spit up (as a baby)
4=Failure to thrive
5=Constipation
6=Diarrhea
7=Swollen abdomen
8=Pale in color
9=Lethargy or weakness
10=Other → Please specify "Other" primary symptom
11=I don't know

In general, how long were symptoms present prior to diagnosis?
1= Under 1 month
2= 1 month or more but less than 6 months
3= 6 months or more but less than 1 year
4= 1 year or more but less than 5 years
5= 5 years or more
6= I did not experience any symptoms

[If under 1 month] Within that one month, how long after symptoms began was the diagnosis confirmed?
1= Within 24 hours of onset of symptoms
2= 2 days after onset of symptoms
3= 3 days or more but less than 1 week after onset of symptoms
4= 1 week after onset of symptoms or more
5=I don't know
In general, how long was the primary symptom present prior to diagnosis?
1= Under 1 month
2= 1 month or more but less than 6 months
3= 6 months or more but less than 1 year
4= 1 year or more but less than 5 years
5= 5 years or more
6= I did not experience any symptoms

[If under 1 month] Within that one month, how long after the primary symptom began was the diagnosis confirmed?
1= Within 24 hours of onset of symptoms
2= 2 days after onset of symptoms
3= 3 days or more but less than 1 week after onset of symptoms
4= 1 week after onset of symptoms or more
5=I don't know

[If parent respondent] Did your child ever have yellow tinted spit up as a newborn while in the hospital shortly after birth? 1=Yes 2=No 3= I don't know

Prior to diagnosis, did you have any of the following due to gastrointestinal symptoms? (Check all that apply) 1=Frequent primary care/pediatrician visits
2=Specialty care visits
3=Urgent care visits
4=Emergency room/department visits

5=Hospitalizations
6=I don't know

[If urgent care] How frequently did you seek urgent care services prior to diagnosis?
1=Rarely 2=A few times 3=Often

[If emergency] How frequently did you go to the emergency room/department prior to diagnosis?
1=Rarely 2=A few times 3=Often

What test procedure(s) were done prior to diagnosis? (check all that apply)
1=Upper GI with small bowel follow-through
2=Upper GI without small bowel follow-through
3=X-ray
4=CT
5=MRI
6=Ultrasound
7=Lower GI/barium enema
8=Endoscopy
9=No test – diagnosed through surgery
10= Other → Please specify "Other" tests procedures
11=I don't know

What test procedure confirmed the diagnosis? (check all that apply)
1=Upper GI with small bowel follow-through
2=Upper GI without small bowel follow-through
3=X-ray
4=CT
5=MRI
6=Ultrasound
7=Lower GI/barium enema
8=Endoscopy
9=No test – diagnosed through surgery
10= Other → Please specify "Other" test that confirmed diagnosis
11=I don't know

What health care professional ordered the diagnostic test?
1=Pediatrician/primary care provider
2=Gastroenterologist
3=Surgeon
4=Emergency
5=Other → Please specify "Other" health care professional
6=I don't know

Was the diagnosis of malrotation with or without volvulus (intestinal twist)?
1=With volvulus
2=Without volvulus
3=I don't know

Was the volvulus determined to be complete or incomplete (partial)?
1=Complete
2=Incomplete (partial)
3=I don't know

What year was the diagnosis of malrotation confirmed?

What month was the diagnosis of malrotation confirmed?

Treatment

This section of the survey asks about you or your child's initial treatment for malrotation following diagnosis.

Did the patient (you or your child) have surgery as a treatment for malrotation?

1=Yes
0=No → *Skip to Nonsurgical Management section*

How has the intestinal malrotation been treated? (check all that apply)

1=Surgical intervention for volvulus
2=Ladd's surgical procedure
3=Alternative intestinal surgery
4=I don't know

<u>**[If surgery for volvulus]**</u>

What year was the initial surgery for volvulus?

What month was the initial surgery for volvulus?

How long of a delay occurred between the identification of volvulus and the start of the surgery?

1=Less than 1 hour
2=More than 1 hour but less than 2 hours
3=More than 2 hours but less than 5 hours
4=More than 5 hours but less than 10 hours
5=More than 10 hours
6=I don't know

<u>**[If Ladd's procedure]**</u>

[If surgery for volvulus and Ladd's] Was the Ladd's procedure performed at the same time as the surgical intervention for volvulus?

[If not part of the surgery for volvulus] What year was the initial Ladd's procedure done?

[If not part of the surgery for volvulus] What month was the initial Ladd's procedure done?

How long of a delay occurred between the malrotation diagnosis and the completion of the Ladd's procedure?

1=Less than 24 hours
2=More than 24 hours but less than 1 week
3=More than 1 week but less than 1 month
4=More than 1 month but less than 6 months
5=More than 6 months
6=I don't know

Was the initial Ladd's performed as an open surgery (laparotomy with large incision) or closed surgery (laparoscopy with small incisions)?

1=Open surgery (large incision)
2=Closed surgery (small incisions)
3=I don't know

Was there a bowel resection (part of bowel removed) as part of the initial Ladd's procedure?

1=Yes 2=No 3= I don't know

[If resection] Did the resection during Ladd's include the removal of small intestine/bowel or large intestine/bowel? (check all that apply)

1=Small intestine/bowel
2=Large intestine/bowel
3=I don't know

[If small bowel] Approximately how much small intestine/bowel was removed? (include number and units - example 5 inches or 10 cm)

[If large bowel] Approximately how much large intestine/bowel was removed? (include number and units - example 5 inches or 10 cm)

Was the appendix removed as part of the Ladd's procedure? 1=Yes 2=No 3= I don't know

For the Ladd's procedure, how many days was the hospital stay?

1=Less than 5 days
2=5 days or more but less than 10
3=10 days or more but less than 20
4=20 days or more
5=I don't know

Besides surgeons, were any other specialists seen during the hospital stay?

1=Gastroenterologist
2=Nutritionist/Dietician
3=Speech pathologist
4=Occupational therapist
5=Other specialist → Please describe "Other" specialist seen in the hospital
6=No other specialists

Were any follow-up visits scheduled with the surgeon at discharge? 1=Yes 2=No 3= I don't know

Before hospital discharge for Ladd's, were there any follow-up visits scheduled with specialists besides surgery at discharge?

1=Gastroenterologist
2=Nutritionist/Dietician
3=Speech pathologist
4=Occupational therapist
5=Other specialist → Please describe "Other" specialist scheduled for follow-up
6=No other specialists

Were any of the following types of medical management present at discharge following the Ladd's procedure? (check all that apply)

1=Ostomy
2=Tube feeding
3=Total parental nutrition (TPN)
4=Specialty formula/nutrition
5=Pain management
6=Other → Please describe "Other" conditions at discharge
7=None of the above

[If no Ladd's] What was/were the reason(s) for not having the Ladd's procedure after diagnosis? (check all that apply)

1= No or minimal symptoms
2= Personal choice to not have surgery
3=Difficulty finding surgeon

4=Costs of surgery/insurance coverage challenges
6=Chose to have alternative surgery instead
5=Other → Please describe "Other" reasons for not having the Ladd's procedure

[If alternative intestinal surgery]

What year was the alternative intestinal procedure done?

What month was the alternative intestinal procedure done?

Was there a bowel resection (part of bowel removed) as part of the alternative intestinal procedure?
1=Yes 2=No 3= I don't know

[If resection] Did the resection during the alternative surgery include the removal of small intestine/bowel or large intestine/bowel? (check all that apply) 1=Small intestine/bowel
2=Large intestine/bowel
3=I don't know

[If small bowel] Approximately how much small intestine/bowel was removed? (include number and units - example 5 inches or 10 cm)

[If large bowel] Approximately how much large intestine/bowel was removed? (include number and units - example 5 inches or 10 cm)

Was the appendix removed as part of the alternative intestinal procedure? 1=Yes 2=No 3= I don't know

For the alternative intestinal surgery, how many days was the hospital stay?
1=Less than 5 days
2=5 days or more but less than 10
3=10 days or more but less than 20
4=20 days or more
5=I don't know

Were any of the following conditions present at discharge following the alternative intestinal surgery?
1=Ostomy
2=Tube feeding
3=Total parental nutrition (TPN)
4=Specialty formula/nutrition
5=Pain management
6=Other → Please describe "Other" conditions at discharge
7=None of the above

Post-surgical Outcomes

This section of the survey asks about you or your child's gastrointestinal health after the malrotation diagnosis and initial surgery for malrotation.

Immediately after having the Ladd's procedure, were there any surgical complications, such as bleeding, wound issues, or hernias? 1=Yes 2=No 3= I don't know

Which of the following surgical complications occurred? (check all that apply)
1=Postoperative hemorrhage (bleeding)
2=Wound infection
3=Wound dehiscence
4=Incisional hernia
5=I don't know

[If Ladd's]

After having the Ladd's procedure, were there any gastrointestinal symptoms present or did symptoms in general go away?

1=Yes, there were still gastrointestinal symptoms present
2=No, there were generally no gastrointestinal symptoms after Ladd's
3=I don't know

[If symptoms] What gastrointestinal symptoms were present after Ladd's? (Check all that apply)

1=Vomiting
2=Abdominal pain
3=Excessive spit up (as a baby)
4=Failure to thrive
5=Constipation
6=Diarrhea
7=Swollen abdomen
8=Reflux
9=Difficulty tolerating foods
10=Other → Please describe "Other" gastrointestinal symptoms present after Ladd's
11=I don't know

[If symptoms] Compared to before Ladd's, were the symptoms after Ladd's:

1=Less severe
2=About the same severity
3=More severe
4=I don't know

[If symptoms] Compared to before Ladd's, were the symptoms after Ladd's:

1=Less frequent
2=About the same frequency
3=More frequent
4=I don't know

How long did symptoms continue following the Ladd's procedure?

1=Up to 3 months
2=More than 3 months but less than 6 months
3= 6 months or more but less than a year
4=1 year or more
5=I don't know

How many different surgeons have you seen for gastrointestinal symptoms?

1=0
2=1
3=2
4=3
5=4 or more
6=I don't know

After surgery, have you seen any of the following specialists? (Check all that apply)

1=Gastroenterologist
2=Nutritionist/Dietician
3=Speech pathologist
4=Occupational therapist
5=Other specialist → Please describe "Other" specialist seen
6=No other specialists

[if Gastroenterologist] How many different gastroenterologists have been seen?

1=1
2=2
3=3
4=4 or more

After having the Ladd's procedure, have any further tests been performed to examine additional symptoms? 1=Yes 2=No 3= I don't know

[If yes] Which of the following procedure have been performed after the Ladd's procedure?

1=Upper GI with small bowel follow-through
2=Upper GI without small bowel follow-through
3=X-ray
4=CT
5=MRI
6=Ultrasound
7=Lower GI/barium enema
8=Endoscopy
9=No test – diagnosed through surgery
10= Other → Please specify "Other" tests procedures
11=I don't know

After having the Ladd's procedure, did any of the following occur? (check all that apply)

1= Emergency room or emergency department visits for gastrointestinal symptoms
2= Additional hospitalizations for gastrointestinal issues
3= Additional abdominal surgery
4=Recurrent volvulus
5=Bowel obstructions
6=None of these occurred after Ladd's

[If emergency] How many emergency room or emergency department visits were there for gastrointestinal symptoms?

1=1 visit
2=2 to 4 visits
3=5 to 9 visits
4=10 or more visits
5=I don't know

[If hospitalizations] How many hospital admissions were there for gastrointestinal symptoms?

1=1 hospitalization
2=2 to 4 hospitalizations
3=5 to 9 hospitalizations
4=10 or more hospitalizations
5=I don't know

[If surgery] How many additional abdominal surgeries have occurred since Ladd's?

1=1 more abdominal surgery
2=2 more abdominal surgeries
3=3 more abdominal surgeries
4=4 or more abdominal surgeries
5=I don't know

[If surgery] Has the Ladd's procedure been repeated since the initial Ladd's procedure?
1=Yes 2=No 3= I don't know

[If additional Ladd's] How many additional Ladd's procedures have been done?
1=One additional Ladd's procedure has been done after the initial Ladd's (2 total)
2=Two additional Ladd's procedure has been done after the initial Ladd's (3 total)
3=Three or more additional Ladd's procedures has been done after the initial Ladd's (4 or more total)

[If additional Ladd's] Were any additional Ladd's procedure performed as an open surgery (laparotomy with large incision) 1=Yes 2=No 3= I don't know

[If recurrent volvulus] How many times has there been a diagnosed recurrence of volvulus?
1= 1 additional recurrences of volvulus (2 total)
2= Two additional recurrences of volvulus (3 total)
3= Three or more additional recurrences of volvulus (4 or more total)

[If surgery] Have any of the additional surgeries after Ladd's resulted in a bowel resection (part of the bowel removed)? 1=Yes 2=No 3= I don't know

[If resection] Did the resections include the removal of small intestine/bowel or large intestine/bowel? (check all that apply)
1=Small intestine/bowel
2=Large intestine/bowel
3=I don't know

[If small bowel] Approximately how much small intestine/bowel was removed? (include number and units - example 5 inches or 10 cm)

[If large bowel] Approximately how much large intestine/bowel was removed? (include number and units - example 5 inches or 10 cm)

Nonsurgical Management

Have you used nonsurgical methods to manage symptoms of malrotation, such as medications, dietary changes, or alternative medicine?
1=Yes
0=No → *Skip to Current Health Status section*

[If nonsurgical methods] Thinking about the first year after diagnosis, what nonsurgical methods were or are used to manage malrotation symptoms? (check all that apply)
1=Medications
2=Dietary changes
3=Physical therapy → Please describe Physical therapy management
4=Alternative medicine → Please describe Alternative medicine
5=Other → Please describe "Other" methods that are used to manage malrotation symptoms
6=No symptoms to manage

[If Medications] Which medications are used to manage symptoms? (check all that apply)
1=Reflux medications
2=Laxatives
3=Antidiarrheal medications
4=Motility medications
5=Pain medications
6=Antispasmodic medications
7=Other

[If Dietary] What dietary changes are used to manage symptoms? (check all that apply)
1=Dairy free

2=Gluten free
3=Soy free
4=Low FODMAP
5=Low Fiber
6=High Fiber
7=Supplemental nutrition/formula
8=Parenteral nutrition (PN or TPN)
9=Other → Please describe "Other" dietary changes

[If TPN] What were the reasons for the use of parenteral nutrition (PN or TPN)? (check all that apply)
1=Short bowel syndrome (removal of bowel requiring PN)
2=Motility issues
3=Severity of pain with eating
4=Intestinal ischemia (loss of blood flow)
5=Other → Please describe "Other" reasons for PN

Current Health Status

This section of the survey asks about you or your child's current gastrointestinal health and quality of life during the past year.

Have you had an ache or pain in your stomach or belly (gut) in the last year? (please do not count cramps or pain with menstrual periods and do not count pain in your chest) 1=Yes 0=No

Have you had this same ache or pain more than SIX times in the past year? 1=Yes 0=No

How bad is the ache or pain usually?
1=Mild: can be ignored if you don't think about it
2=Moderate: cannot be ignored, but does not affect your life-style
3=Severe: affects your lifestyle
4=Very severe: markedly affects your lifestyle

Does your usual ache or pain ever wake you from sleep at night? 1=Yes 0=No

Does this pain come and go periodically? Periodically here means periods of at least a month with no pain, with periods in between of weeks to months when there is pain. 1=Yes 0=No

How many times did you get this pain in the last year? (Check one answer)
1=Less than once a month
2=About once a month
3=About once a week
4=Several times a week
5=Daily

When this pain occurs, how long does it usually last? (Check one answer)
1=Less than 30 minutes
2=30 minutes to 2 hours
3=More than 2 hours to 6 hours
4=More than 6 hours

Has your bowel habit changed in the last year? 1=Yes 0=No

How would you describe your usual bowel pattern in the last year (Check one answer)
1=Normal
2=Constipated

3=Diarrhea
4=Alternating constipation and diarrhea

Do you take anything (e.g. bran, fiber, laxatives) because of constipation? 1=Yes 0=No

What do you take and how often?

Have you seen mucus in your stools in the last year (that is, white or green slimy material)? 1=Yes 0=No

How many times have you had a feeling of WANTING to throw up (nausea) in the last year?
1=None
2=Less than once a month
3=About once a month
4=About once a week
5=Several times a week
6=Daily

How many times have you ACTUALLY thrown up (vomited) in the last year?
1=None
2=Less than once a month
3=About once a month
4=About once a week
5=Several times a week
6=Daily

Do you often feel bloated and actually see your belly swell up? 1=Yes 0=No

Have you often had difficulty swallowing (food sticking in your throat) in the last year? 1=Yes 0=No

Have you had HEARTBURN (a burning or ache behind the breast bone in the chest) in the last year? Do not count pain from angina or heart trouble)
1=None
2=Less than once a month
3=About once a month
4=About once a week
5=Several times a week
6=Daily

Family History and Additional Diagnoses

Is there any other family member who has been diagnosed with malrotation? 1=Yes 2=No 3= I don't know

[If Yes] When considering the patient with malrotation (either you or your child), what family member(s) have been diagnosed with malrotation? (Check all that apply)
1=The patient's parent
2= The patient's grandparent
3= The patient's sibling
4= The patient's child
5= The patient's extended relative (aunt, uncle, cousin)
6=I don't know

Has the patient (either you or your child) ever been diagnosed with any of the following other gastrointestinal conditions in addition to malrotation? (Check all that apply)
1=Gastroesophageal reflux disease (GERD)

2=Functional constipation
3=Irritable Bowel Syndrome (IBS)
4=Crohn's Disease
5=Ulcerative Colitis
6=Hirschprung's Disease
7=Achalasia
8=Eosinophilic esophagitis
9=Cyclic Vomiting Syndrome (CVS)
10=Dysmotility
11=Gastroparesis
12=Intestinal pseudo-obstruction
13=Intussusception
14=Short Bowel Syndrome
15=Gastroschisis
16=Imperforate anus
17=Meckel Diverticulum
18=Omphalocele
19=Pyloric Stenosis
20=Duodenal Atresia
21=Congenital diaphragmatic hernia
22=Other gastrointestinal condition → Please describe "Other gastrointestinal condtion"

Has the patient (either you or your child) ever been diagnosed with any of the following other conditions? (Check all that apply)
1=Kidney or ureter absence
2=Polysplenia (multiple accessory spleens)
3=Asplenia (absent spleen)
4=Biliary atresia
5=Heterotaxy
6=Other cardiac or heart anomaly → Please describe "Other cardiac or heart anomaly"
7=Chiari malformation
8=Ehlers-Danlos Syndrome (EDS)
9=Postural Orthostatic Tachycardia Syndrome (POTS)
10=Dysautonomia
11=Other condition → Please describe "Other condition"

Other Information

On a scale of 1 to 5 with 1 being very dissatisfied and 5 being very satisfied

How do you feel about the overall health care you have received for intestinal malrotation?
1=Very dissatisfied
2=Dissatisfied
3=Neither dissatisfied or satisfied
4=Satisfied
5=Very satisfied

How do you feel about the primary care / pediatric care (if applicable) you have received for intestinal malrotation?
1=Very dissatisfied
2=Dissatisfied
3=Neither dissatisfied or satisfied
4=Satisfied
5=Very satisfied

How do you feel about the surgical consultation and/or care you have received for intestinal malrotation?

1=Very dissatisfied
2=Dissatisfied
3=Neither dissatisfied or satisfied
4=Satisfied
5=Very satisfied

How do you feel about the follow-up gastrointestinal care you have received for intestinal malrotation?

1=Very dissatisfied
2=Dissatisfied
3=Neither dissatisfied or satisfied
4=Satisfied
5=Very satisfied

How far do you generally have to travel to receive follow-up care for intestinal malrotation/gastrointestinal issues?

1= Do not currently receive follow-up care for intestinal malrotation/gastrointestinal issues.
2= Less than a 30 minute drive
3=More than a 30 minute drive but under an hour
4=More than a 1 hour drive but under 3 hours
5=More than a 3 hour drive but under 6 hours
6=More than a 6 hour drive but usually drive to receive care
7= Usually fly to receive care for intestinal malrotation

Have you ever searched for help for malrotation or gastrointestinal issues beyond your local doctors or health care system?
1=Yes 0=No

Have you ever traveled out of state for consultation or care related to intestinal malrotation/gastrointestinal issues? 1=Yes 0=No

How likely would you <u>currently</u> be willing to travel to a national center that specializes in treating malrotation?

1=Not at all likely
2=Somewhat likely
3=Very likely

If in the future your gastrointestinal symptoms returned or worsened, how likely would you be willing to travel to a national center that specializes in treating malrotation?

1=Not at all likely
2=Somewhat likely
3=Very likely

The IMPOWER Registry includes six month update surveys. Do we have your permission to contact you in 6 months? 1=Yes 0=No

What is the best email address to contact you for the registry updates?

What is a secondary email address we can use to contact you for the registry updates?

What is your phone number?

What is a secondary phone number we can try to reach you?

May we contact you in the future for other research study opportunities for malrotation separate from the 6-month registry updates? 1=Yes 0=No

Is there anything else that you feel important to share that has not already been covered?

**Patient Demographics Updates (Every 6 months)**

Thank you for completing a 6 month follow-up survey for the IMPOWER Registry. First, we would like to get your updated contact information. This section of the survey asks about you or your child's current gastrointestinal health and quality of life IN THE LAST 6 MONTHS.

Have you had an ache or pain in your stomach or belly (gut) in the six months? (please do not count cramps or pain with menstrual periods and do not count pain in your chest) 1=Yes 0=No

Have you had this same ache or pain more than THREE times in the past year? 1=Yes 0=No

How bad is the ache or pain usually?

1=Mild: can be ignored if you don't think about it
2=Moderate: cannot be ignored, but does not affect your life-style
3=Severe: affects your lifestyle
4=Very severe: markedly affects your lifestyle

Does your usual ache or pain ever wake you from sleep at night? 1=Yes 0=No

Does this pain come and go periodically? Periodically here means periods of at least a month with no pain, with periods in between of weeks to months when there is pain. 1=Yes 0=No

How many times did you get this pain in the last 6 months? (Check one answer)

1=Less than once a month
2=About once a month
3=About once a week
4=Several times a week
5=Daily

When this pain occurs, how long does it usually last? (Check one answer)

1=Less than 30 minutes
2=30 minutes to 2 hours
3=More than 2 hours to 6 hours
4=More than 6 hours

Has your bowel habit changed in the last 6 months? 1=Yes 0=No
How would you describe your usual bowel pattern in the last 6 months (Check one answer)

1=Normal
2=Constipated
3=Diarrhea
4=Alternating constipation and diarrhea

Do you take anything (e.g. bran, fiber, laxatives) because of constipation? 1=Yes 0=No

What do you take and how often?

Have you seen mucus in your stools in the last 6 months (that is, white or green slimy material)? 1=Yes 0=No

How many times have you had a feeling of WANTING to throw up (nausea) in the last 6 months?

1=None
2=Less than once a month
3=About once a month
4=About once a week
5=Several times a week
6=Daily

How many times have you ACTUALLY thrown up (vomited) in the last 6 months?
1=None
2=Less than once a month
3=About once a month
4=About once a week
5=Several times a week
6=Daily

Do you often feel bloated and actually see your belly swell up? 1=Yes 0=No

Have you often had difficulty swallowing (food sticking in your throat) in the last 6 months? 1=Yes 0=No

Have you had HEARTBURN (a burning or ache behind the breast bone in the chest) in the last 6 months? Do not count pain from angina or heart trouble
1=None
2=Less than once a month
3=About once a month
4=About once a week
5=Several times a week
6=Daily

Family History and Additional Diagnoses Updates

In the last 6 months, has there been any other family member who has been diagnosed with malrotation? 1=Yes 2=No 3= I don't know

When considering the patient with malrotation (either you or your child), what family member(s) has been diagnosed with malrotation in the last 6 months? (Check all that apply)
1=The patient's parent
2= The patient's grandparent
3= The patient's sibling
4= The patient's child
5= The patient's extended relative (aunt, uncle, cousin)
6=I don't know

Did the patient (either you or your child) have any conditions diagnosed in the last 6 months?
1=Yes 0=No

Which of the following diagnoses were made in the last 6 months? (Check all that apply)
1=Gastroesophageal reflux disease (GERD)
2=Functional constipation
3=Irritable Bowel Syndrome (IBS)
4=Crohn's Disease
5=Ulcerative Colitis
6=Hirschprung's Disease
7=Achalasia
8=Eosinophilic esophagitis
9=Cyclic Vomiting Syndrome (CVS)

10=Dysmotility
11=Gastroparesis
12=Intestinal pseudo-obstruction
13=Intussusception
14=Short Bowel Syndrome
15=Meckel Diverticulum
16=Pyloric Stenosis
17=Duodenal Atresia
18=Kidney or ureter absence
19=Polysplenia (multiple accessory spleens)
20=Asplenia (absent spleen)
21=Biliary atresia
22=Heterotaxy
23=Other cardiac or heart anomaly → Please describe "Other cardiac or heart anomaly"
24=Chiari malformation
25=Ehlers-Danlos Syndrome (EDS)
26=Postural Orthostatic Tachycardia Syndrome (POTS)
27=Dysautonomia
28=Other condition → Please describe "Other condition"

The IMPOWER Registry includes six month update surveys. Do we have your permission to contact you in 6 months? 1=Yes 0=No

Is there anything else that you feel important to share that has not already been covered?
