# Supplementary material for: IMPOWER: a national patient-generated registry for intestinal malrotation exploring diagnosis, treatment, and surgical outcomes
Source: Orphanet J Rare Dis. 2023 May 11;18:113. doi: 10.1186/s13023-023-02722-5 (PMC10176693; doi:10.1186/s13023-023-02722-5)
Supplement: Supplementary file 2 — Additional file 2. Tables S1–S4. This file presents data from the adult registrants diagnosed before 20 years of age in comparison to the pediatric registrants and the adult registrants diagnosed after 20 years of age. [file 13023_2023_2722_MOESM2_ESM.pdf]

Supplemental Table 1. Participant Demographics and Clinical Characteristics in IMPOWER[a]

Characteristic	Pediatric registrants[b] (n=119)	Adult registrants diagnosed before 20 years of age (n=17)	Adult registrants diagnosed after 20 years of age (n=55)
**Female, n(%)**	52 (43.7)	13 (76.5)	51 (92.7)
**White Race, n(%)**	110 (94.0)	17 (100.0)	55 (100.0)
**Region**			
Northeast	18 (15.1)	5 (29.4)	14 (25.4)
Midwest	28 (23.5)	5 (29.4)	17 (30.9)
South	46 (38.7)	7 (41.2)	15 (27.3)
West	15 (12.6)	0 (0.0)	9 (16.4)
**Age at Diagnosis, n(%)**			
<1 month	68 (57.1)	7 (41.2)	-
1 month to <1 year	22 (18.4)	1 (5.9)	-
1 year to <5 years	15 (12.6)	0 (0.0)	-
5 years to <10 years	9 (7.6)	0 (0.0)	-
10 years to <20 years	4 (3.4)	9 (52.9)	-
20 years or more	-	-	55 (100.0)
**Symptoms present prior to diagnosis, n(%)**	106 (89.1)	16 (94.1)	54 (98.2)
**Time from symptoms to diagnosis, n(%)**			
Under 1 month	65 (54.6)	7 (41.2)	6 (10.9)
1 month or more to less than 6 months	22 (18.5)	0 (0.0)	6 (10.9)
6 months or more to less than 1 year	2 (1.7)	3 (17.6)	3 (5.5)
1 year to less than 5 years	10 (8.4)	0 (0.0)	8 (14.5)
5 years or more	6 (5.0)	6 (35.3)	31 (56.4)
No symptoms or unknown	14 (11.7)	1 (5.9)	1 (1.8)
**All symptoms reported prior to diagnosis, n(%)**			
Vomiting	86 (72.3)	11 (64.7)	28 (50.9)
Excessing spit up as a baby	64 (53.8)	7 (41.2)	7 (12.7)
Lethargy/weakness	47 (39.5)	6 (35.3)	28 (50.9)
Other	44 (37.0)	5 (29.4)	22 (40.0)
Abdominal pain	43 (36.1)	9 (52.9)	51 (92.7)
Constipation	38 (31.9)	8 (47.1)	37 (67.3)
Swollen abdomen	34 (28.6)	4 (23.5)	31 (56.4)
Pallor/pale in color	32 (26.9)	5 (29.4)	12 (21.8)
Failure to thrive	26 (21.8)	2 (11.8)	7 (12.7)
Diarrhea	13 (10.9)	4 (23.5)	27 (49.1)
**Primary symptom at diagnosis, n(%)**			
Vomiting	46 (38.7)	8 (47.1)	5 (9.1)
Excessing spit up as a baby	18 (12.6)	2 (11.8)	0 (0.0)
Lethargy/weakness	3 (2.5)	0 (0.0)	1 (1.8)
Other	18 (15.1)	1 (5.9)	6 (10.9)
Abdominal pain	10 (8.4)	4 (23.5)	36 (65.5)
Constipation	1 (0.8)	1 (5.9)	1 (1.8)
Swollen abdomen	8 (6.7)	0 (0.0)	3 (5.5)
Pallor/pale in color	1 (0.8)	0 (0.0)	0 (0.0)
Failure to thrive	3 (2.5)	0 (0.0)	0 (0.0)
Diarrhea	1 (0.8)	0 (0.0)	2 (3.6)
**Volvulus at diagnosis, n(%)**			
Yes	83 (69.7)	9 (52.9)	15 (27.3)
No	28 (23.5)	5 (29.4)	34 (61.8)
**Green or Yellow Colored Emesis, n(%)**			
Vomit/Spit Up Ever Green	55 (46.2)	5 (29.4)	12 (21.8)
Vomit/Spit Up Ever Yellow	70 (58.8)	8 (47.1)	24 (43.6)
Vomit/Spit Up Ever Red/Brown	12 (10.1)	0 (0.0)	4 (7.3)
Yellow spit up shortly after birth	52 (43.7)	-	-
**Pain Constant or Intermittent, n(%)**			
Constant	7 (5.9)	1 (5.9)	9 (16.4)
Intermittent	29 (24.4)	9 (52.9)	42 (76.4)
No pain or unknown	83 (69.8)	7 (41.2)	4 (7.3)

[a]Intestinal Malrotation Patient Outcomes and WEllness Registry
[b]Pediatric registrants under 18 at time of enrollment were registered by parent or caregiver

Supplemental Table 2. Health Care Visits and Testing until Diagnosis among Participants in IMPOWER[a]

Characteristic	Pediatric registrants[b] (n=119)	Adult registrants diagnosed before 20 years of age (n=17)	Adult registrants diagnosed after 20 years of age (n=55)
**Healthcare visits prior to diagnosis**			
Frequent primary care/pediatrician visits	53 (44.5)	7 (41.2)	29 (52.7)
Specialty care visits	23 (19.3)	4 (23.5)	28 (50.9)
Urgent care visits	18 (15.1)	5 (29.4)	15 (27.3)
Emergency department visits	42 (35.3)	7 (41.2)	34 (61.8)
Hospitalizations	27 (22.7)	6 (35.3)	10 (18.2)
None of the above or unknown	40 (33.6)	7 (41.2)	7 (12.7)
**All tests leading up to diagnosis**			
Upper GI with small bowel follow through	63 (52.9)	10 (58.8)	24 (43.6)
X-ray	79 (66.4)	10 (58.8)	28 (50.9)
Ultrasound	48 (40.3)	9 (52.9)	29 (52.7)
CT	32 (26.9)	7 (41.2)	49 (89.1)
Upper GI	25 (21.0)	3 (17.6)	12 (21.8)
Lower GI/barium enema	16 (13.4)	0 (0.0)	11 (20.0)
MRI	15 (12.6)	5 (29.4)	17 (30.9)
Swallow study	14 (11.8)	1 (5.9)	6 (10.9)
Endoscopy	8 (6.7)	4 (23.5)	27 (49.1)
Gastric emptying study	7 (5.9)	0 (0.0)	12 (21.8)
Other test	14 (11.7)	1 (5.9)	13 (23.6)
No test, through surgery or autopsy	14 (11.8)	9 (52.9)	2 (3.6)
**Test that confirmed diagnosis**			
Upper GI with small bowel follow through	49 (41.2)	8 (47.1)	16 (29.1)
X-ray	11 (9.2)	1 (5.9)	2 (3.6)
Ultrasound	6 (5.0)	1 (5.9)	0 (0.0)
CT	13 (10.9)	2 (11.8)	38 (69.1)
Upper GI	11 (9.2)	1 (5.9)	0 (0.0)
Lower GI/barium enema	6 (5.0)	0 (0.0)	0 (0.0)
MRI	3 (2.5)	0 (0.0)	3 (5.5)
Swallow study	4 (3.4)	0 (0.0)	1 (1.8)
Endoscopy	1 (0.8)	0 (0.0)	1 (1.8)
Gastric emptying study	0 (0.0)	0 (0.0)	1 (1.8)
Other test	8 (6.7)	2 (0.0)	0 (0.0)
No test, through surgery or autopsy	23 (19.3)	1 (0.0)	5 (9.1)
**Clinician that ordered confirmation test**			
Emergency Department clinician	33 (27.7)	3 (17.6)	17 (30.9)
Surgeon	22 (18.5)	3 (17.6)	7 (12.7)
Intensive care unit (ICU) clinician	16 (13.4)	0 (0.0)	0 (0.0)
Primary care/pediatrician	15 (12.6)	5 (29.4)	10 (18.2)
Gastroenterologist	15 (12.6)	3 (17.6)	19 (34.5)
Other health care provider	10 (8.4)	5 (29.4)	0 (0.0)
Unknown	5 (6.9)	6 (35.3)	2 (3.6)
**Surgical procedure for malrotation and/or volvulus**	107 (89.9)	16 (94.1)	45 (81.8)

[a]Intestinal Malrotation Patient Outcomes and WEllness Registry
[b]Pediatric registrants under 18 at time of enrollment were registered by parent or caregiver

Supplemental Table 3. Post-Surgical Outcomes among Surgical Participants in IMPOWER[a]

Characteristic, n(%)	Pediatric registrants[bc] (n=107)	Adult registrants diagnosed before 20 years of age (n=16)	Adult registrants diagnosed after 20 years of age (n=45)
**Urgency of surgical procedure**			
Emergency to preserve life	73 (68.2)	9 (56.3)	13 (28.9)
Urgent within 48 hours	18 (16.8)	1 (6.3)	5 (11.1)
Recommended within next month	11 (10.3)	4 (25.0)	18 (40.0)
Elective or presented as a choice	2 (1.9)	0 (0.0)	9 (20.0)
**Bowel resection during initial surgical procedure**	34 (31.8)	4 (25.0)	10 (22.2)
**Short bowel diagnosis after surgical procedure**	25 (23.4)	1 (6.3)	3 (6.7)
**Length of hospital stay at initial surgical procedure**			
Under 1 day / outpatient	0 (0.0)	1 (6.3)	5 (11.1)
1 day or more but less than 3 days	7 (6.2)	2 (12.5)	10 (22.2)
3 days or more but less than 5 days	11 (9.7)	0 (0.0)	5 (11.1)
5 days or more but less than 10 days	32 (29.9)	4 (25.0)	13 (28.9)
10 days or more but less than 20 days	23 (21.5)	2 (12.5)	10 (22.2)
20 days or more	33 (30.8)	4 (25.0)	1 (2.2)
**Postsurgical complications**	17 (15.9)	3 (18.8)	13 (28.9)
**Outcomes after initial surgical procedure**			
Emergency department visits	52 (48.6)	7 (43.8)	27 (60.0)
Additional hospitalizations	35 (32.7)	8 (50.0)	15 (33.3)
Additional abdominal surgical procedure	27 (25.2)	6 (37.5)	14 (31.1)
Bowel obstruction	17 (15.9)	5 (31.3)	12 (26.7)
Volvulus or recurrent volvulus	3 (2.8)	3 (18.8)	3 (6.67)
**Number of additional surgical procedures**			
1 additional procedure	11 (10.3)	3 (18.8)	5 (11.1)
2 additional procedures	7 (6.5)	2 (12.5)	5 (11.1)
3 or more additional procedures	9 (8.4)	1 (6.3)	4 (8.9)

[a]Intestinal Malrotation Patient Outcomes and WEllness Registry
[b]Pediatric participants under 18 at time of enrollment were registered by parent or caregiver
[c]Excludes participants deceased at time of registration

Supplemental Table 4. Ongoing Gastrointestinal (GI) Symptoms among Surgical Participants 1 Year Post Operation in IMPOWER[a]

Characteristic, n(%)	Pediatric registrants[bc] (n=107)	Adult registrants diagnosed before 20 years of age (n=16)	Adult registrants diagnosed after 20 years of age (n=45)
**GI symptoms reported 1 year following surgical procedure**			
Yes	67 (62.6)	7 (43.8)	37 (82.2)
No	38 (35.5)	8 (50.0)	7 (15.6)
**Type of GI symptoms 1 year following surgical procedure**			
Vomiting	39 (36.4)	4 (25.0)	11 (24.4)
Difficulty tolerating foods	36 (33.6)	6 (37.5)	19 (42.2)
Reflux	35 (32.7)	5 (31.3)	9 (20.0)
Abdominal pain	34 (31.8)	6 (37.5)	33 (73.3)
Constipation	30 (28.0)	6 (37.5)	22 (48.9)
Diarrhea	28 (26.2)	1 (6.3)	16 (35.6)
Excessive spit up (baby)	26 (24.3)	0 (0.0)	--
Swollen abdomen	26 (24.3)	1 (6.3)	13 (28.9)
Failure to thrive	21 (19.6)	1 (6.3)	5 (11.1)
Other	10 (9.3)	1 (0.0)	11 (24.4)
**Severity of symptoms 1 year following surgical procedure**			
More severe	7 (6.5)	1 (6.3)	10 (27.0)
About the same severity	9 (8.4)	3 (18.8)	9 (24.3)
Less severe	37 (34.6)	3 (18.8)	17 (46.0)
Generally no symptoms prior to surgical procedure	46 (43.0)	8 (50.0)	1 (2.7)
**Frequency of symptoms 1 year following surgical procedure**			
More frequent	10 (9.3)	3 (18.8)	13 (35.1)
About the same frequency	18 (16.8)	2 (12.5)	8 (21.6)
Less frequent	25 (23.4)	2 (12.5)	14 (37.8)
Generally no symptoms prior to surgical procedure	47 (43.9)	8 (50.0)	1 (2.7)
**Specialist visits following surgical procedure**			
Gastroenterologist	74 (69.2)	12 (75.5)	37 (82.2)
Nutritionist/Dietician	45 (42.1)	4 (25.0)	16 (35.6)
Physical therapist	29 (27.1)	2 (12.5)	5 (11.1)
Occupational therapist	27 (25.2)	1 (6.3)	--
Speech pathologist	25 (23.4)	0 (0.0)	--
Feeding therapist	22 (20.6)	0 (0.0)	--
Lactation specialist	17 (15.9)	0 (0.0)	--
Allergist	12 (11.2)	0 (0.0)	2 (4.4)
Other specialist	19 (17.8)	1 (6.3)	4 (8.9)
**Number of gastroenterologists seen following surgical procedure**			
0 gastroenterologists	33 (30.8)	4 (25.0)	8 (17.8)
1 gastroenterologist	20 (18.7)	3 18.8)	10 (22.2)
2 gastroenterologists	27 (25.2)	4 (25.0)	8 (17.8)
3 gastroenterologists	9 (8.4)	1 (6.3)	3 (6.7)
4 or more gastroenterologists	18 (16.8)	3 (18.8)	16 (35.6)
**Nonsurgical methods currently used to manage symptoms**			
Medications	28 (26.2)	9 (56.3)	32 (71.1)
Dietary changes	27 (25.2)	8 (50.0)	31 (68.9)
Alternative medicine	6 (5.6)	1 (6.3)	10 (22.2)
Physical therapy	3 (2.8)	0 (0.0)	10 (22.2)
Other nonsurgical methods	12 (11.2)	0 (0.0)	3 (6.7)

[a]Intestinal Malrotation Patient Outcomes and WEllness Registry
[b]Pediatric participants under 18 at time of enrollment were registered by parent or caregiver
[c]Excludes participants deceased at time of registration
